# Supplementary material for: The Rickettsia actin-based motility effectors RickA and Sca2 contribute differently to cell-to-cell spread and pathogenicity
Source: mBio. 2025 Jan 17;16(2):e02563-24. doi: 10.1128/mbio.02563-24 (PMC11796396; doi:10.1128/mbio.02563-24)
Supplement: Supplemental Figures — Figures S1 to S3. [file mbio.02563-24-s0002.pdf]

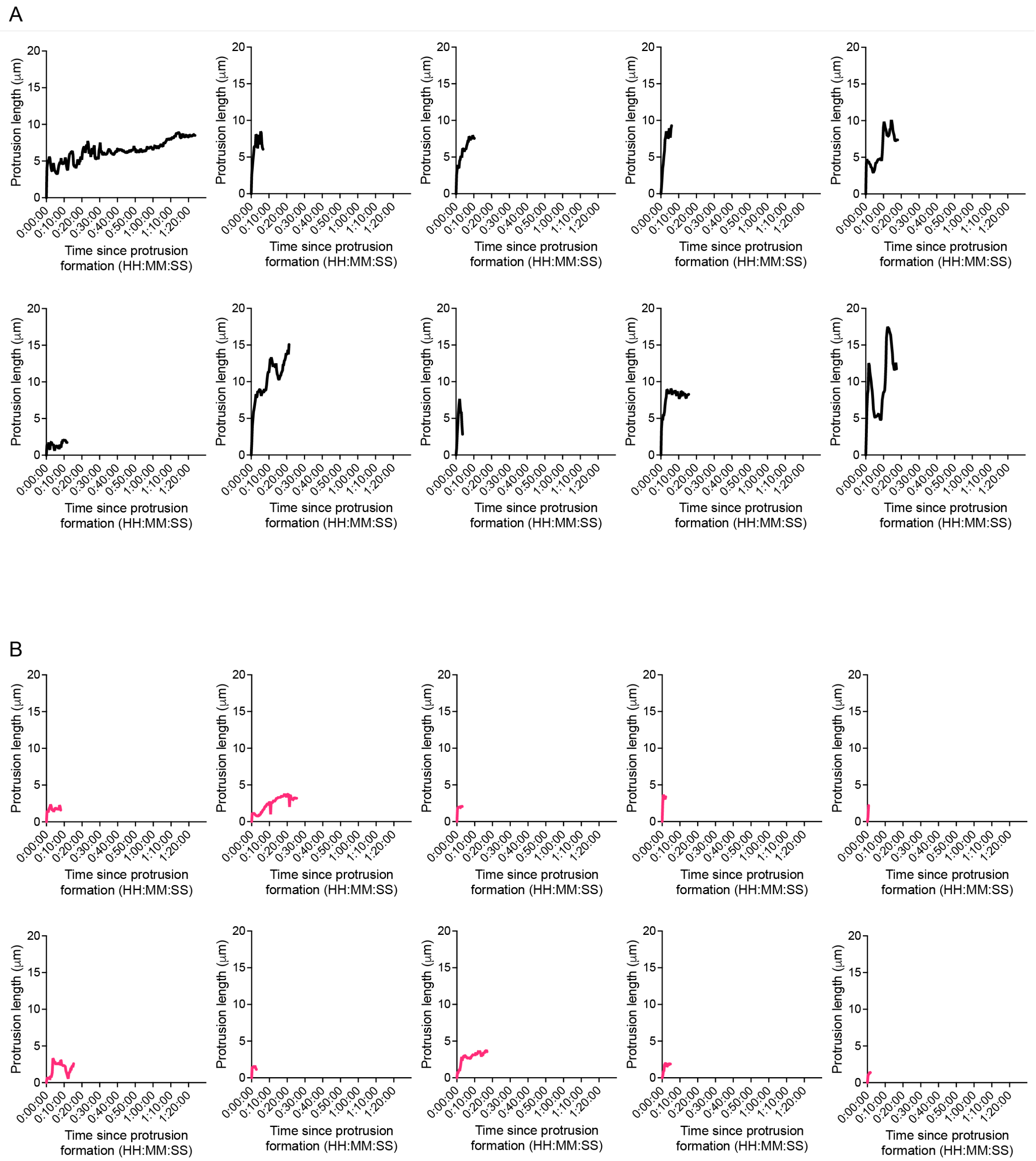

Figure S1, related to Figure 4 – Protrusion lengths during individual spread events.

Each graph shows the length of an individual protrusion over time during **(A)** RickA-spread (*sca2*::Tn mutant) or **(B)** Sca2-spread (*rickA*::Tn mutant). These data are included in Figure 4b.

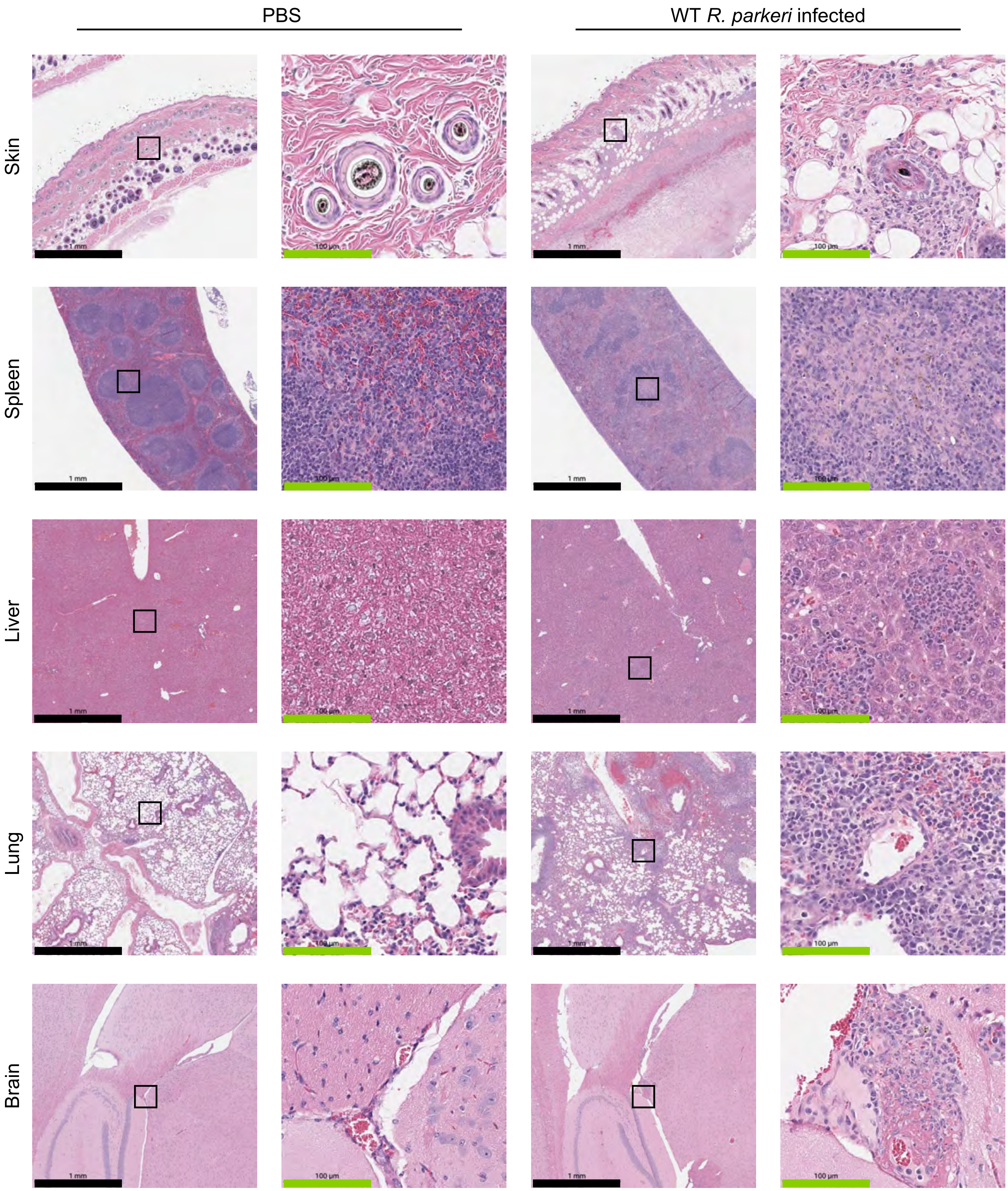

Figure S2, related to Figure 7 – H&E staining of various organs.

Representative images of H&E staining of the indicated organs in mice either injected i.d. with PBS (control) or infected with 10^3 pfu WT *R. parkeri*. Results were qualitatively similar for *rickA*::Tn-infected and *sca2*::Tn-

infected mice. Black squares on the left were magnified on the right. Black scale bars represent 1 mm, green scale bars represent 100 μm.

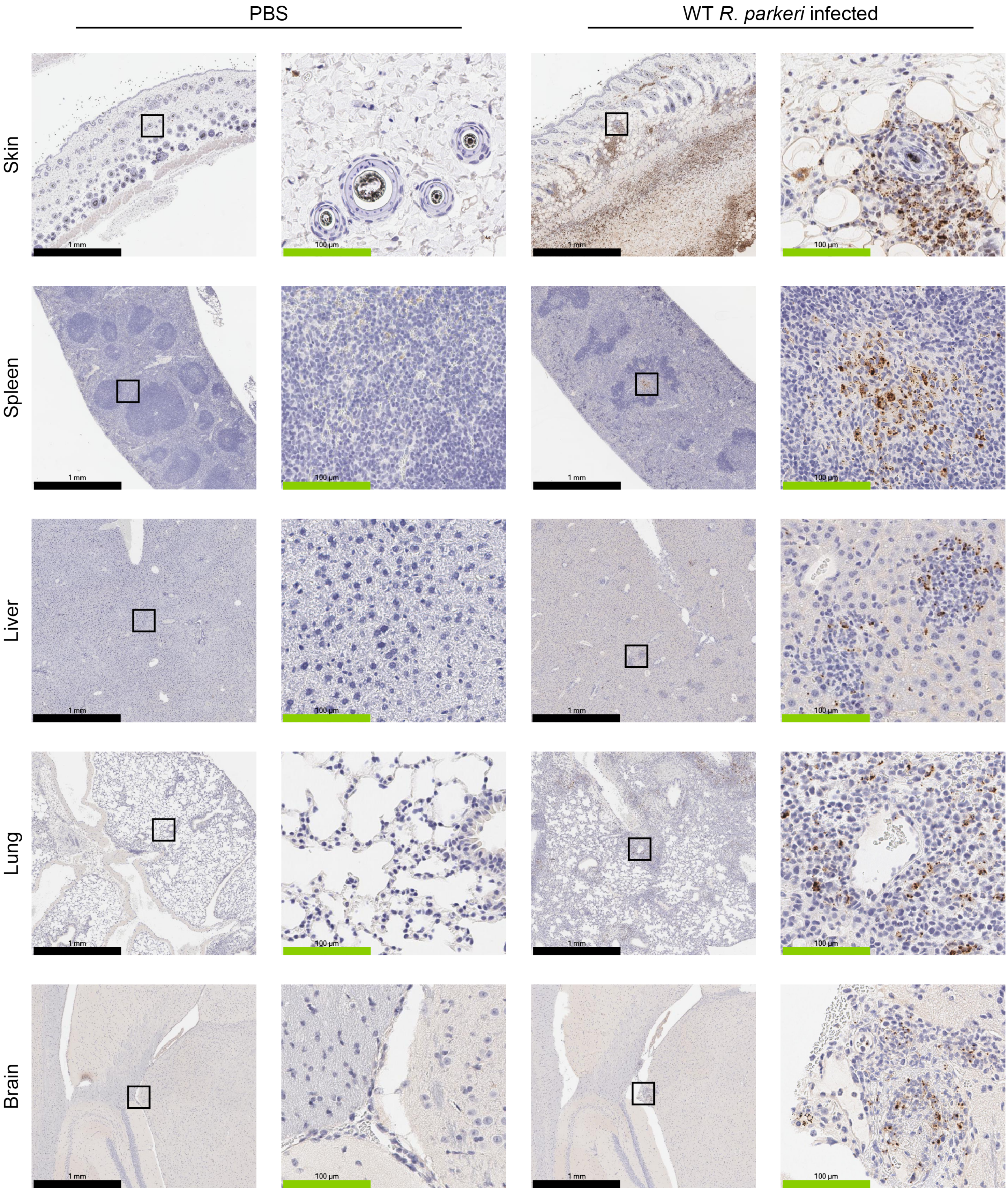

Figure S3, related to Figure 7 – IHC staining of various organs.

Representative images of anti-*Rickettsia* IHC staining of the indicated organs in mice either injected i.d. with PBS (control) or infected with 10^3 pfu WT *R. parkeri*. Results were qualitatively similar for *rickA*::Tn-infected

and *sca2*::Tn-infected mice. Black squares on the left were magnified on the right. Black scale bars represent 1 mm, green scale bars represent 100 μm.
