# Supplementary material for: The Rickettsia actin-based motility effectors RickA and Sca2 contribute differently to cell-to-cell spread and pathogenicity
Source: mBio. 2025 Jan 17;16(2):e02563-24. doi: 10.1128/mbio.02563-24 (PMC11796396; doi:10.1128/mbio.02563-24)
Supplement: Supplemental Legends — Legends for Movies S1 to S4 and source data. [file mbio.02563-24-s0003.docx]

**Movie S1, related to Figure 2 – RickA-motility mediates to cell-to-cell spread.**

Timelapse imaging of *sca2*::Tn mutant *R. parkeri* (cyan) undergoing RickA-motility and spreading from cell-to-cell following infection of A549 cells that express the F-actin marker Lifeact-3xTagBFP (grey) and the plasma membrane marker TagRFP-t-farnesyl (magenta). Cells were imaged every 20 s starting at 5-10 min post infection. Yellow arrowhead indicates the bacterium of interest. Yellow line indicates the approximate boundary between cells. Video is shown at 5 frames/s. Timestamps are MM:SS. Scale bar is 5 µm.

**Movie S2, related to Figure 2 – RickA-motility can lead to unsuccessful spread events.**

Timelapse imaging of *sca2*::Tn mutant *R. parkeri* (cyan) undergoing RickA-motility and failing to spread from cell-to-cell following infection of A549 cells that express the F-actin marker Lifeact-3xTagBFP (grey) and the plasma membrane marker TagRFP-t-farnesyl (magenta). Cells were imaged every 20 s starting at 5-10 min post infection. Yellow arrowhead indicates the bacterium of interest. Video is shown at 5 frames/s. Timestamps are MM:SS. Scale bar is 5 µm.

**Movie S3, related to Figure 3 – Sca2-motility mediates cell-to-cell spread.**

Timelapse imaging of *rickA*::Tn mutant *R. parkeri* (cyan) undergoing Sca2-motility and spreading from cell-to-cell following infection of A549 cells that express the F-actin marker Lifeact-3xTagBFP (grey) and the plasma membrane marker TagRFP-t-farnesyl (magenta). Cells were imaged every 20 s starting at ~28 h post infection. Yellow arrowhead indicates the bacterium of interest. Green square indicates the area being magnified. Video is shown at 5 frames/s. Timestamps are HH:MM:SS. Scale bar is 5 µm.

**Movie S4, related to Figures 4 and S1 – Protrusions oscillate between elongation and retraction.**

Timelapse imaging of *sca2*::Tn mutant *R. parkeri* (cyan) undergoing RickA-motility and spreading from cell-to-cell following infection of A549 cells that express the F-actin marker Lifeact-3xTagBFP (grey) and the plasma membrane marker TagRFP-t-farnesyl (magenta). Cells were imaged every 20 s starting at 5-10 min post infection. Yellow arrowhead indicates the bacterium of interest. Video is shown at 5 frames/s. Timestamps are MM:SS. Scale bar is 5 µm.

**Source Data.**

Spreadsheet containing source data for Fig. 1C, 1E, 4A-G, 5A-C, 6A-E, 7A-E, 7G-K, and 8A.
